# Relationship between early age at menarche, older age at menopause and subtypes of breast cancer: a scoping review

**DOI:** 10.61622/rbgo/2024rbgo50

**Published:** 2024-09-06

**Authors:** Lucas Casagrande Passoni Lopes, Gabriel Araújo Medeiros, Igor José Nogueira Gualberto, Thales Baptista Gut, Rafael Vasconcelos Silva Ferrazini, Carlos Antonio Negrato

**Affiliations:** 1 Faculdade de Medicina Universidade de São Paulo Bauru SP Brazil Faculdade de Medicina, Universidade de São Paulo, Bauru, SP, Brazil.; 2 Faculdade de Medicina Universidade de São Paulo São Paulo SP Brazil Faculdade de Medicina, Universidade de São Paulo, São Paulo, SP, Brazil.

**Keywords:** Breast neoplasms, Menarche, Menopause, Aged, Age factors

## Abstract

**Objective:**

To determine the relationship between early age at menarche, late age at menopause with specific subtypes of breast cancer (BC).

**Methods:**

A literature search was conducted in Embase, Lilacs, PubMed, Scopus, and Scielo databases, following the Joanna Briggs Institute scoping review protocol and answering the question “How early age at menarche or late age at menopause are related to different breast cancer subtypes?”.

**Results:**

A number of 4,003 studies were identified, of which 17 were selected. Most of the included articles found a clear relationship between early menarche, late menopause and some subtypes of BC, mainly, PR+, ER+, luminal, and HER-2 tumors. However, some studies have found a contradictory relationship and one study didn’t find any relationship between them.

**Conclusion:**

A relationship between early age at menarche and advanced age at menopause was observed with some subtypes of breast cancer, since other factors must be considered in its understanding.

## Introduction

Breast cancer (BC) is the second most common type of cancer found among women in Western countries, after non-melanoma skin cancer.^1^ It is rare before the age of 35, but its incidence grows rapidly and progressively with aging, being most commonly diagnosed between 40 and 60 years. BC presents a lifetime risk of 12.4% and is the most frequent cancer-related cause of death in women worldwide, representing a significant public health issue in all countries.^2,3)^

Breast cancer results from an interplay between biological, environmental, and genetic factors, such as aging, short breastfeeding periods, family history, nulliparity, late age at first birth, postmenopausal hormone replacement therapy, hysterectomy in late menopause, the expression of the XRCC3 gene and high body mass index (BMI).^2,4,5^ Early age at menarche and older age at menopause, have also been pointed as risk factors for the development of BC due to extended exposition to steroid hormones, such as estrogens.^4,6-8^ It should be noted that the ages at menarche and menopause vary among individuals and are also influenced by complex social, environmental, and genetic factors.^2,9^ Each 1-year delay in menopause increases the risk of BC by 3%, and each 1-year delay in menarche decreases the risk of BC by 5%.^2,10^

Classifying BC is crucial for defining its prognosis and treatment. The World Health Organization (WHO) proposes histological and molecular classifications. Regarding histological aspects, several subtypes of BC can be found, being ductal (in situ and invasive) and lobular (in situ and invasive) carcinomas are the most prevalent. Molecular classification is made by the expression of some surface molecules, such as estrogen receptors (ER), progesterone receptors (PR) and the human epidermal growth factor receptor 2 (HER2), that may be present or not in different subtypes of BC. Also, the degree of expression of the cellular proliferation biomarker Ki-67 is considered. Consequently, BC can be classified as luminal A (ER+ and/or PR+, HER2 - and low expression of Ki-67), luminal B (ER+ and/or PR+, HER2- and high expression of Ki-67), HER2 (ER-, PR- and HER2+) and triple negative (ER-, PR- and HER2-).^11^

This scoping review aims to map and synthesize the relationship between early age at menarche and late age at menopause with the risk of specific subtypes of BC.

## Methods

The present study is a scoping literature review, which allows a broad and comprehensive view of records on a respective topic and enables the synthesis of relevant evidence that addresses and informs clinical practice and identifies existing gaps in knowledge. This study follows the proposal of the Joanna Briggs Institute.^12^

Eligible studies were those performed with premenopausal or postmenopausal women with the diagnosis of BC, that contained data regarding age at menarche and age at menopause. After a detailed search, we found multiple subtypes of classifications, as well as multiple age thresholds for early/late menarche and early/late menopause.

The Population, Concept, and Context (PCC) strategy was used to formulate the guiding research question and the search strategy. Thus, we defined “P” as women with BC; “C” as the relationship between early age at menarche or late age at menopause with different BC subtypes; and the last “C” as data found in articles published in the previous ten years. Considering this definition, the following guiding question was formulated: “How early age at menarche or late age at menopause are related to different breast cancer subtypes?”. The included articles had to contain the three elements of the PCC strategy, answer the research question, and be written in English, Portuguese or Spanish, from January 1st, 2013, until October 20th, 2022. Articles written in other languages, in different time frames, that did not answer the guiding question, case reports, congress abstracts, book chapters, guidelines, roundups, expert opinions, brochures, published in non-indexed sources and reviews were excluded. However, the inclusion criteria were modified as the researchers became more acquainted with the available evidence, as this is possible in scoping reviews.^13^ After a second look at all selected articles for full-text analysis, the exclusion criteria for being review articles was reanalyzed. Some manuscripts initially labeled as reviews were, in reality, reanalyses of cohort, case-only, or case-control data. After careful consideration, we concluded that some articles contained unique sample data and differed from traditional reviews. They presented the results of particular studies exclusively, with geographical and timespan uniqueness. Thus, avoiding the potential bias arising from data duplication if all review articles were included, we designated these studies as “exclusive reanalysis” for inclusion criteria.

The search for articles was conducted between the 15th and 19th of October, 2022 in the following databases: National Library of Medicine (PubMed/MEDLINE), Embase, SCOPUS, Web Of Science, SciElo, and Latin American and Caribbean Health Sciences Literature (LILACS/BVS). For the search, health descriptors (Decs/Mesh), keywords, and their alternative terms, and the Boolean operators OR and AND were used in all databases as presented in chart 1.

**Chart 1:** Databases and search descriptors selected according to PCC strategy

Database	Search strategies
MEDLINE	(“Menopause”) AND (“breast neoplasms” OR “Breast Cancer” OR “Breast Carcinoma” OR “Breast Malignant Neoplasm” OR “Breast Malignant Tumor” OR “Cancer of Breast” OR “Cancer of the Breast” OR “Breast Tumor”) AND (“Menarche”)
WEB OF SCIENCE	TS=(Menopause AND (breast neoplasms OR Breast Cancer OR Breast Carcinoma OR Breast Malignant Neoplasm OR Breast Malignant Tumor OR Cancer of Breast OR Cancer of the Breast OR Breast Tumor) AND Menarche)
LILACS AND SciElo	(Menopause) AND (breast neoplasms) OR (Breast Cancer) OR (Breast Carcinoma) OR (Breast Malignant Neoplasm) OR (Breast Malignant Tumor) OR (Cancer of Breast) OR (Cancer of the Breast) OR (Breast Tumor) AND (Menarche)
SCOPUS	(TITLE-ABS-KEY ( “Menopause” AND ( “Breast neoplasms” OR “Breast Cancer” OR “Breast Malignant Neoplasm” OR “Breast Malignant Tumor” ) AND “Menarche” ))
Embase	((‘menopause’/exp OR menopause) AND (‘breast neoplasms’/exp OR ‘breast neoplasms’ OR ‘breast cancer’/exp OR ‘breast cancer’ OR ‘breast malignant neoplasm’ OR ‘breast malignant tumor’) AND (‘menarche’/exp OR menarche))

The selection process was performed by three independent reviewers. Disagreements were resolved through consensus.

As our purpose had a scoping nature, the risk of bias assessing eligible studies was not considered mandatory.^14^

## Results

Based on the initial search, 4,003 articles were identified. Out of this, 2,585 articles were removed for not being published in the last ten years, and 747 were duplicates and were also removed with the support of Mendeley reference manager. Another 440 articles were excluded, for being published in non-indexed journals, 18 were not written in English, Spanish, or Portuguese, 103 were not the selected subtype for analysis, one was a case report, and 318 did not answer the guiding question.

**Figure 1:**
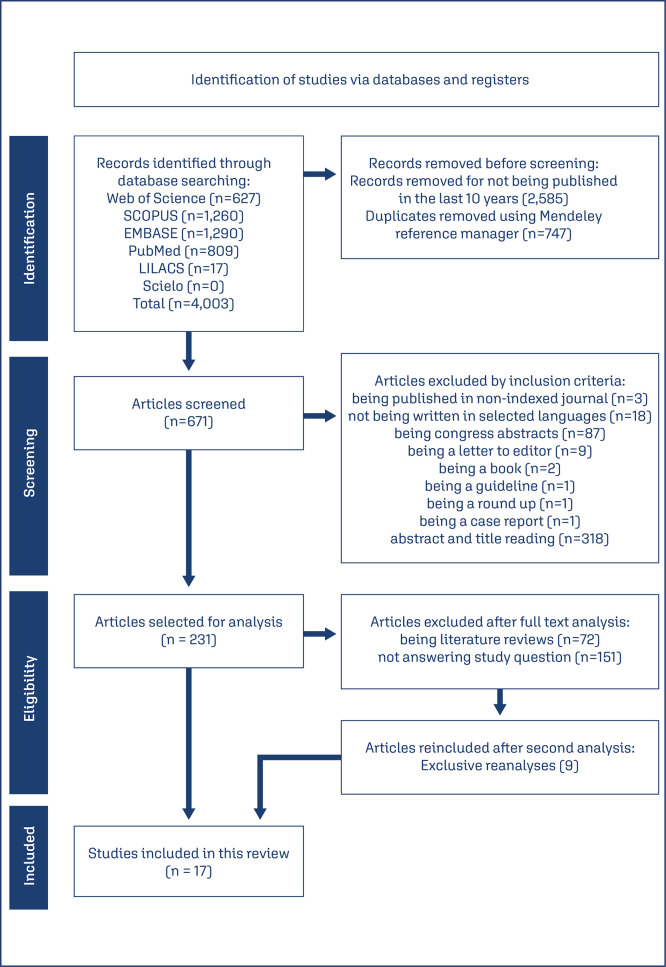
Flowchart of the study selection process, adapted from PRISMA-ScR

Thus, a total of 231 articles were screened for full-text reading and analysis, 151 were excluded for answering only partially the guiding question, and 72 were excluded for being literature reviews, of which nine were reincluded as “exclusive reanalysis” after a second look for eligibility. 17 articles remained as the final sample, as shown in figure 1.

The results are presented in the form of tables and discursive reports. To comply with the methodological rigor, the PRISMA extension tool adapted for scoping reviews was applied.^14^

**Figure 2:**
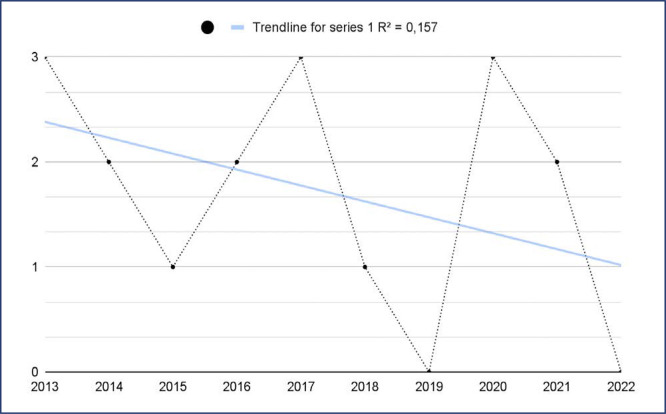
Trendline of year distribution of articles

Of the 17 included studies, three were conducted in 2013, two in 2014, one in 2015, two in 2016, three in 2017, one in 2018, three in 2020 and two in 2021, as shown in figure 2.^2,5,8,15-28^

The selected studies were conducted in various sites worldwide. 18 countries contributed to the results. Most studies took place in the United States (n = 7), followed by Italy, Mexico, Norway, Turkey (n = 2), China, Denmark, France, Germany, Greece, Israel, Indonesia, Kenya, Puerto Rico, Spain, Sweden, The Netherlands and United Kingdom (n = 1). Some studies were conducted in only one country (Indonesia, Israel, Kenya, Mexico, USA, Turkey), or multiple countries.

The most commonly used research designs were case-control (n = 7), but there were also cohort (N = 5) and cross-sectional studies (n = 5).

Among the 17 included studies, sample sizes varied from 108 to 30, 680 participants. The populations also differed regarding age and ethnicity, which included different nationalities and ethnicities between countries and within the same country, such as African-Americans and Latin Americans in the USA. The age range could not be specified since the studies did not describe minimum and maximum values.

The characteristics of the selected articles can be seen in chart 2 (which describes the name of the authors, year of publication, country and study design).

**Chart 2:** Description of the included studies according to authors, year of publication, country, study design, overall population, Breast cancer molecular subtype, Main results and conclusions, definitions of age at menarche and age at menopause

Authors/year	Country	Study design	Overall population	Breast cancer molecular subtype	Main results and conclusions	Definition of “Age at menarche” and “Age at menopause”
Martinez et al. (2013)^(15)^	Mexico, United States of America	Exclusive cross-sectional reanalysis	1,041	HER2+, triple-negative, luminal A	Earlier age at menopause was found to be associated with an incidence of triple-negative tumors, which are typically more aggressive than other subtypes. - The study did not find any significant association between age at menarche and breast cancer subtypes. Overall, the study suggests that reproductive factors may play a role in the development of different breast cancer subtypes in Mexican-American women	Early menarche (<13 y); Late menarche (≥13 y); Early menopause (<50 y); Late menopause (≥50 y)
Ritte et al. (2013)^(16)^	Germany Denmark, France, Italy, Greece, Norway, Spain, Sweden, The Netherlands and United Kingdom	Exclusive prospective cohort reanalysis	4,565	ER+PR+ and ER-PR-	Both HR+ and HR- breast cancers have similar association with early menarche. However, HR- type association was weak and not statistically significant. - The study also found that age at menopause showed no significant association with either HR+ or HR- tumor subtypes.	Early menarche (<13 y); Late menarche (≥15 y); Early menopause (≤48 y) Late menopause (≥55 y)
Turkoz et al. (2013)^(17)^	Turkey	Cross-sectional	1,884	Luminal A, Luminal B, HER-2 overexpressing, Triple negative	There were no significant differences between risk of breast cancer subtypes and early menarche and late menopause	Early menarche (<12 y); Late menarche (≥12 y); Early menopause (<55 y); Late menopause (≥55 y)
Cui et al. (2014)^(18)^	United States of America	Exclusive population-based case-control reanalysis	4,172	ER+ and ER-	- Late menarche (age at menarche ≥ 14 years) was significantly associated with reduced risk of ER+ breast cancer, but not significantly associated with ER- tumors. These associations were seen in Whites, but not in African- Americans. - Compared to women with early menopause (< 40 years), those whose age at menopause was 47-51 years had higher risk for both ER+ and ER- tumors	Early menarche (≤11 y); Late menarche (≥14 y); Early menopause (< 40 y); Late menopause (≥52 y)
Rosato et al. (2014)^(19)^	Italy	Exclusive case–control reanalysis	2,552	Isolated ER -, Isolated ER +, ER-PR-, ER-PR+, ER+PR- and ER+PR+	- No significant association was found between the age at menarche and breast cancer risk either in ER- or ER+ breast cancers. - A stronger association in ER+ than that in ER- breast cancers was observed for older age at menopause. - Significant associations were found in ER+PR+ and isolated ER+ breast cancers for older age at menopause	Early menarche (<13 y); Late menarche (≥15 y); Early menopause (<50 y) Late menopause (≥50 y)
O’Brien et al. (2015)^(20)^	United States of America and Puerto Rico	Exclusive case-control reanalysis	3,054		Late menarche, early menopause, may protect against invasive breast cancer in young women. We observed overall concordance between risk factors for young-onset DCIS and invasive breast cancer	Early menarche (<12 y); Late menarche (≥14 y); Early menopause (<45 y); Late menopause (≥45 y)
Chen et al. (2016)^(8)^	United States of America	Exclusive cross-sectional reanalysis	2,710	Luminal A (ER+/HER2-), luminal B (ER+/HER2+), triple negative (TN, ER-/PR-/HER2-), and HER2-overexpressing (H2E, ER-/HER2+)	Age at menopause was positively associated with odds of TN breast cancer. - Age at menopause was reported to be only positively associated with luminal cancers relative to cancer-free controls with no differences in risks seen across case subtypes. - No differences across breast cancer subtypes were observed with age at menarche	Early menarche (<12 y); Late menarche (≥14 y); Early menopause (<45 y); Late menopause (≥55 y)
Sisti et al. (2016)^(21)^	United States of America	Prospective cohort	3,768	Luminal A (ER+/HER2-), luminal B (ER+/HER2+), triple negative (TN, ER-/PR-/HER2-), and HER2-overexpressing (H2E, ER-/HER2+)	- Age at menopause was positively associated with risk of luminal A, luminal B and unclassified tumors, and suggestively associated with HER2-enriched tumors, though no association with basal-like was observed. - The inverse association with age at menarche was not heterogeneous across subtypes, though a significant trend was only observed with luminal A. - There was no significant heterogeneity across breast cancer subtypes for most reproductive risk factors, though many appeared most strongly associated with luminal A tumors, including ages at menarche and menopause.	Early menarche (<12 y); Late menarche (>14 y); Menopause not defined
Ellingjord-Dale et al. (2017)^(22)^	Norway, United Kingdom and United States of America	Exclusive population-based case-control reanalysis	30,680	Luminal A-like, luminal B-like HER2-negative, luminal B-like HER2-positive, HER2-positive, and triple-negative	- Age at menopause was positively associated with overall breast cancer risk (i.e. all subtypes combined) whereas age at menarche was associated with a decreased risk. - Age at menopause, comparing women >52 to women <47 years old, was positively associated with risk of luminal B-like HER2-positive (ER+ PR+/PR- HER2+) and HER2-positive (ER- PR- HER2+) subtypes	Early menarche (≥9 and ≤12 y); Late menarche (≥15 and ≤18 y); Early menopause (<47 y); Late menopause (>52 y)
Li et al. (2017)^(5)^	China and United States of America	Case-control	2,672	Luminal (ER+ or PR+, HER2+ or HER2-), HER2-enriched (ER-, PR-, HER2+), or triple-negative (ER-, PR-, HER2-)	- Late menarche was negatively associated with luminal tumor risk, while late menopause increased risk of all subtypes of breast cancer	Early menarche (≤13 y); Late menarche (≥15 y); Menopause not defined
Mullooly et al. (2017)^(23)^	United states of America	Exclusive population-based prospective cohort reanalysis	10,355		- Younger age at menopause was associated with a higher risk of ductal carcinoma *in situ*, but with a lower risk of invasive ductal carcinoma. Similar to our findings, significant heterogeneity was not observed between the risks of ductal carcinoma *in situ* and invasive ductal carcinoma for most breast cancer risk factors, including age at menarche	Early menarche ( ≤12 y) and late menarche (≥15 y); Early menopause (<45 y) and late menopause (≥55 y)
Soewoto et al. (2018)^(24)^	Indonesia	Cross-sectional	108	ER+, ER-	-The longer the estradiol exposure of 39.89 years indicates the presence of high grade (grade III) breast cancer, p-value <0.05	Early menarche (≤12 y); Late menarche (>12 y); Early menopause (<45 y); Late menopause (>60 y)
John et al. (2020)^(25)^	United States of America	Exclusive population-based case-control reanalysis	14,209	HR+, HR-	Older age at menarche was associated with reduced risk of both HR+ and HR- breast cancer. For HR+, later menarche and earlier menopause were associated with lower risk in non-Hispanic Whites and Hispanics. For HR-, lower risk was associated with later menarche, except in African- Americans and older Asian- Americans	Early menarche (<12 y); Late menarche (≥14 y); Early menopause (≤45 y); Late menopause (≥51 y)
Rojas-Lima et al. (2020)^(26)^	Mexico	Case-Control	1,539	HR+/HER2- (ER+ and/or PR+ and HER2−), HER2+(HR+ or HR−), and TN (HR− and HER2−).	Age at menopause was significantly higher in HR+/HER2− and HER2+ cases. Higher age at menarche was negatively associated only with HR+/HER2− tumors. No associations of TN tumors were found with age at menarche and at menopause	Early menarche (≤12 y); Late menarche (≥15 y); Early menopause (<43 y); Late menopause (≥48 y)
Ulgen et al. (2020)^(27)^	Turkey	Retrospective cohort	211	ER+/-, PR+/- and HER2+/-	Early age at menarche was less common in the ER-/PR- group than in the ER+/PR+ group	Early menarche (≤12 y)
Korzets et al. (2021)^(2)^	Israel	Retrospective cohort	620	ER+/PR+/HER2- and ER+/PR-/HER2-	Factors that increase endogenous estrogen exposure, such as early menarche and late menopause, are associated with increased risk of breast cancer, particularly ER-positive. Early age at menarche was associated with PR positivity. Menopause was associated with PR negativity.	Early menarche (<12 y); Late menarche (≥12 y); Early menopause (<45 y) Late menopause (>55 y)
Sayed et al. (2021)^(28)^	Kenya	Cross-sectional	821	ER-	Older age at menarche was associated with ER-negative patients in older but not in younger women. Additionally, HER2-enriched patients were less likely to be obese and had older age at menopause	Early menarche (<13 y); Late menarche (≥13 y); Early menopause (<50 y); Late menopause (≥50 y)

Most of the studies explored molecular subtypes,^2,5,8,15-19,21,22,24-28^ while a minority of them discussed histological classifications.^20,23,24^ Notably, there were variations in the definitions of menarche and menopause across the studies, especially concerning the specific age thresholds used to classify early and late occurrence of these events. Additionally, some studies did not provide an explicit definition of menopause,^5,21,27^ while one did not specify what was considered a late menarche.^27^ Some highlights are pointed out below.

- Early menarche was commonly defined as that occurring before the age of 13 in most studies;^(15,16,19,22-24,26-28)^
- Late menarche definition varied across the studies, with age ranges varying from 12 to 15 years and above;
- Early menopause was typically defined as that occurring before the age of 48,^(2,8,16,18,20,22-26)^ although some studies adopted ages ranging from 50 to 55;^(15,17,19,28)^
- Late menopause was generally defined as that occurring at or after the age of 50, though some studies set the threshold at 45, 55, or even 60 years.
- These discrepancies highlight the diversity in methodologies used regarding age at menarche and menopause.

### Breast cancer classification

In general, the studies included in this review covering histological subtypes of BC followed strictly the 2019 WHO classification of BC and the studies concerning molecular subtypes often follow this same classification as seen in Chart 3. However, in many cases, some articles define tumors by the absence or presence of surface proteins such as ER+, PR-, HER2+.

**Chart 3:** Molecular classification of breast cancer

Tumor type	Hormonal receptors (HR)		Protein receptor (HER)	Biomarker Ki-67
	Estrogen Receptor (ER)	Progesterone receptor (PR)		
Luminal A	Present (+)	Present (+)	Absent (-)	Low expression
Luminal B	Present (+)	Present (+)	Absent (-)	High Expression
HER-2	Absent (-)	Absent (-)	Present (+)	-
Triple negative	Absent (-)	Absent (-)	Absent (-)	-

### Relationships observed

Between the included articles, some studies have found a relationship between BC subtypes only with early age at menarche, or only with late age at menopause, but the majority of them have found a relationship between BC subtypes with both, early age at menarche and with late age at menopause.^2,5,8,15,16,18-34^ Finally, only one did not find a relationship between BC subtypes with the age at menarche or age at menopause.^17^

An earlier age at menarche has been related to a higher risk of PR+/ER+ tumors, luminal tumors, and lobular tumors. In Parallel, one study showed that an early age at menarche was a risk factor for presenting BC earlier and with a worse prognosis, while another one, showed that a late age at menarche would be protective against BC, but none of them mentioned a specific subtype of BC. Furthermore, some studies have shown that late age at menarche was related to a reduced risk for ER+ BC only, ER+PR+, Luminal A, Luminal B, HR+/HER2- and for triple negative. Finally, this same late age at menarche was related to a higher risk of ER-PR- and ER+PR+ tumors, ER- tumors and HR+/HR- tumors according to other studies.^2,5,16,18-20,22,24,26 -28,30-32,34^One study has found that a later age at menopause represents a higher risk for developing BC, but does not relate this event with any specific subtype of BC. However, other studies have found a relationship between late age at menopause with greater risk of ER+, ER-, PR+, PR -, Luminal B, Luminal A, HER2+, HER2- and triple negative; moreover, late age at menarche was related with a reduced risk of HER2. An earlier age at menopause was related to a higher risk of triple negative BC by one study, a reduced risk only for ductal carcinoma *in situ* by another and a minor risk for all histological subtypes of BC, except for ductal carcinoma *in situ*.^2,5,8,15,18-26,28,33,34^

## Discussion

Most of the analyzed studies have found a relationship between early age at menarche and late age at menopause with certain subtypes of BC, mainly, PR+, ER+, luminal, and HER-2 subtypes of tumors. However, some studies have found a contradictory relationship between these events and BC subtypes, and one study did not find any relationship.

Finding a relationship between early age at menarche and late age at menopause with some BC subtypes was an expected result since a longer exposure to estrogen could imply a greater risk of BC, as previously described in many studies, including a systematic review and meta-analysis conducted in 2012. For sure, this search found that all BC subtypes, with slight variations among them, occurred more frequently in patients who had an earlier age at menarche and more advanced age at menopause.^6^

Nonetheless, didn’t find a relationship or found contradictory relationships between the earlier age at menarche, late age at menopause and some BC subtypes, which was unexpected. This could be due to the presence of confounding factors that would influence more or less importantly the risk of developing BC.

Indeed, there are some non-modifiable risk factors such as the presence of BRCA1 and BRCA2 genes and also modifiable risk factors, such as environmental exposure to carcinogens, psychological stress and tobacco that can show adverse effects through hormonal and non-hormonal pathways, determining variations in the ages of the first and last menstruation and then, through direct and indirect effects, influence the development of BC.^35-37^

It is also important to consider the role of locoregional factors on the ages at menarche and menopause and the risk of developing BC. For sure, different prevalences of BC were observed between different developed and developing countries, which may be related to nutritional factors, access to medical care, prevention policies, better therapies and even parity.^38,39^

Another fact to be highlighted is the diversity found regarding the definition of early age at menarche and late age at menopause. Indeed, there isn’t a specific age of early menarche and of late menopause defined in the literature, since they vary with temporal and social factors.^40,41^ In this way, our study found ages of early menarche and late menopause that varied from 9 to 13 years and 45 to 60 years, respectively, which could have been confounding factors in establishing the relationship between these events and the risk of developing BC.

Ethnicity has also been shown to be related to the risk of developing certain subtypes of BC.^25^ African-American women who had earlier ages at menarche and late ages at menopause, have been found to have a higher risk of developing ER-, PR- and triple negative tumors.^18^Non-Hispanic Whites and Hispanic American women, who had a late age at menarche and an earlier age at menopause showed a reduced risk of HR+ and HR - BC subtypes.^25^

A great heterogeneity in the classification of BC subtypes between the analyzed studies was also found. Some studies mentioned only 2 and others up to twelve subtypes. Furthermore, most studies mentioned only molecular subtypes of BC, minimizing the discussion on histological subtypes. Thus, despite the existence of a very detailed classification, as the one proposed by the WHO; in general, the studies adapted the classification of subtypes to their scope of analysis and discussion, making comparisons and correlations between the studies difficult.

An important limitation of our study was that data identification underwent reformulations throughout the review process, which may have introduced some bias. However, we believe that the articles designated as “exclusive reanalysis” would be wasted although they are relevant to the objective of this review, which was the reason why they were included. Regarding data extraction, most studies were nonspecific, consequently, some results remained not fully analyzed by the original authors, thus limiting the establishment of relevant relationships.

## Conclusion

A relationship between early age at menarche and advanced age at menopause was observed with some subtypes of BC, since factors such as genes, ethnicity, environmental and locoregional conditions influence this relationship and must be considered in its interpretation.

y, years; BMI, Body Mass Index; HR, Hormone receptor, ER, Estrogen Receptor, PR, Progesterone receptor, HER2, Protein receptor; DCIS, Ductal Carcinoma in situ; LCIS, Lobular Carcinoma in situ, Luminal A, ER and PR present, without HER, low expression of Ki-67; Luminal B, ER and PR present, without HER, high expression of Ki-67; HER-2, ER and PR absent; TN, Triple Negative, ER, PR and HER absent*;* Stage III, higher grade of tumor that still resectable.
